# Out-of-pocket costs for paediatric admissions in district hospitals in Kenya

**DOI:** 10.1111/j.1365-3156.2012.03029.x

**Published:** 2012-06-21

**Authors:** Edwine W Barasa, Philip Ayieko, Susan Cleary, Mike English

**Affiliations:** 1 KEMRI Centre for Geographic Medicine Research – Coast, and Wellcome Trust Research ProgrammeNairobi, Kenya; 2 Health Economics Unit, University of Cape TownCape Town, South Africa; 3 Department of Pediatrics, University of OxfordOxford, UK

**Keywords:** user fees, out-of-pocket costs, child health, hospitals

## Abstract

**Objective:**

To describe out-of-pocket costs of inpatient care for children under 5 years of age in district hospitals in Kenya.

**Methods:**

A total of 256 caretakers of admitted children were interviewed in 2-week surveys conducted in eight hospitals in four provinces in Kenya. Caretakers were asked to report care seeking behaviour and expenditure related to accessing inpatient care. Family socio-economic status was assessed through reported expenditure in the previous month.

**Results:**

Seventy eight percent of caretakers were required to pay user charges to access inpatient care for children. User charges (mean, US$ 8.1; 95% CI, 6.4–9.7) were 59% of total out-of-pocket costs, while transport costs (mean, US$ 4.9; 95% CI, 3.9–6.0) and medicine costs (mean, US$ 0.7; 95% CI, 0.5–1.0) were 36% and 5%, respectively. The mean total out-of-pocket cost per paediatric admission was US$ 14.1 (95% CI, 11.9–16.2). Out-of-pocket expenditures on health were catastrophic for 25.4% (95% CI, 18.4–33.3) of caretakers interviewed. Out-of-pocket expenditures were regressive, with a greater burden being experienced by households with lower socio-economic status.

**Conclusion:**

Despite a policy of user fee exemption for children under 5 years of age in Kenya, our findings show that high unofficial user fees are still charged in district hospitals. Financing mechanisms that will offer financial risk protection to children seeking care need to be developed to remove barriers to child survival.

## Introduction

Access to hospital care plays an important role in improving child survival (Schellenberg *et al.* 2004), and costs have been identified as a significant barrier to access (Perkins *et al.* 2009). In Kenya, public hospitals operate on a cost-sharing arrangement, where the government provides healthcare services at a subsidised rate, financed by central government budgetary allocations to health and supplemented by out-of-pocket payments from users (Management science for health 2001). The cost-sharing policy provides for exemptions of user charges for children under the age of 5 (Carrin *et al.* 2007). Experience in developing countries shows that policies on removal of user fees or exemptions are often poorly implemented (Chuma *et al.* 2009). We examined out-of-pocket payments incurred by caretakers arising from inpatient episodes and explored their magnitude in relation to household expenditures on essential items so as to determine whether out-of-pocket expenditures were catastrophic.

## Methods

The study was conducted in 2008 as part of a larger study to improve the quality of paediatric inpatient care in district hospitals in Kenya (Ayieko *et al.* 2011). Data were collected prospectively, in a survey over 2 weeks, by administering a questionnaire to caretakers of children admitted in eight district hospitals in Kenya. The sampling and selection of hospitals has been described elsewhere (Ayieko *et al.* 2011). We interviewed 256 caretakers (range, 23–36 per hospital). For this descriptive analysis, the data from the eight hospitals were pooled. Total out-of-pocket costs were defined as the sum of transport costs to and from the hospital, user fees and any charges levied for medicines and laboratory services. Caregivers were asked to recall household expenditures they incurred in the previous month. Households were considered to have incurred catastrophic expenditures if their total out-of-pocket costs exceeded 40% of their monthly non-subsistence expenditure (Xu *et al.* 2003). Households were grouped into socio-economic quintiles (1 = lowest to 5 = highest) and two broader groups, higher and lower socio-economic status, based on household monthly expenditures. Out-of-pocket costs and household expenditures were converted from Kenya shillings to US dollars and inflated to 2010 prices using GDP deflators for Kenya. The data were skewed, and so, we explored presenting them as medians and interquartile ranges. Household expenditure categories (rent, food and education) and out-of-pocket cost categories (medicine, transport and user charges) were heavily zero-inflated and hence could only meaningfully be presented as means. Total out-of-pocket costs are presented as both means and medians with non-parametric tests used to test for associations.

## Results

The characteristics of the admitted children are shown in Table 1. The median time of the journey to the hospital by caregivers and the sick children under their care was 45 min (IQR, 30–105). 79.7% (95% CI, 74.2–84.5) of the caregivers used public means to get to hospital while 18.3% (95% CI, 13.7–23.7) walked to the hospital and 2.0% (95% CI, 0.6–4.6) used private means. The median number of visits to a healthcare provider before the child was admitted was 3 (IQR, 1–6).

**Table 1:** Characteristics of admitted children

Age	(*n*) Observations	Median (IQR)
Median age in months	184	11.6 (5.2–24.9)
		Proportion (95% CI)
Gender		
Male	(249) 128	51.4% (45.0–57.8)
Female	(249) 121	48.6% (42.2–56.0)
Diagnosis		
Malaria	(256) 162	63.3% (57.1–69.2)
Pneumonia	(256) 124	40.6% (34.6–46.9)
Diarrhoea and dehydration	(256) 72	28.1% (23.4–34.9)
Other	(256) 24	9.38% (6.10–13.63)
Socio-economic status		
Higher socio-economic group	(250) 123	49.2% (42.8–55.8)
Lower socio-economic group	(250) 127	50.8% (44.4–57.2)
Employment status of caregivers		
Formal employment	(251) 60	23.9% (18.8–29.7)
Informal/unemployed	(251) 191	76.1% (70.3–81.2)

**Figure 1:**
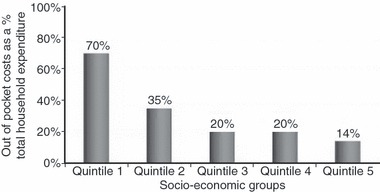
Relationship between out-of-pocket expenditure burden and socio-economic status. (Social-economic status is represented by socio-economic quintiles: Quintile 1 represents the lowest socio-economic group while 5 represents the highest).

Household monthly expenditure on food was 79% of the total monthly expenditure on essentials (food, rent and education); expenditures on rent constituted 11%, and on education 10% (Table 2). The majority of caretakers interviewed (77.7% (95% CI, 72.1–82.7)) reported paying user fees for admission care of sick children. 66.7% (95% CI, 59.3–73.4) used their savings to meet hospital out-of-pocket costs, and 33.3% (95% CI, 26.6–40.7) borrowed money. User charges contributed to the greatest proportion (59%) of out-of-pocket costs, followed by transport (36%) and medicine (5%). Table 3 shows hospitalization expenditure. Figure 1 shows the relationship between out-of-pocket expenditures and their burden (out-of-pocket expenditure as a percentage of total household expenditure) and socio-economic quintiles. Non-parametric tests showed that the household out-of-pocket costs were significantly higher in lower socio-economic strata households (median US$ 11.0 (IQR (4.7–19.3)) than in higher socio-economic strata households (median, US$ 7.0 (IQR, 2.6–14.2)) (rank sum *P* = 0.025) but did not vary significantly with diagnosis. Out-of-pocket costs were catastrophic for 25.4% (95% CI, 18.4–33.3) of households. The proportion of catastrophic health expenditures was higher in households in the lower socio-economic group (41.3% (95% CI, 29.0%–54.4%)) than in the higher socio-economic group (11.5% (95% CI, 5.4%–20.7%)).

**Table 2:** Household monthly expenditures

Expenditure category	Number of caretakers	As% of total expenditure	Mean expenditure US$ (95% CI)
Food*	250	79	56.2 (52.0–60.5)
Education	181	10	6.8 (4.5–9.2)
Rent	154	11	8.2 (3.8–12.5)
Total expenditure	250		66.2 (60.7–71.7)

* Does not include self-produced food.

**Table 3:** Out-of-pocket expenditures associated with inpatient care for children

Cost category	Observations	Median US$ (IQR)	Mean US$ (95% CI)	As % of total
Transport costs	246		4.9 (3.9–6.1)	36
Medicine costs	254		0.7 (0.5–1.0)	5
User charges	254		8.1 (6.4–9.7)	59
Total in-patient out-of-pocket costs	244	7.9 (3.8–17.2)	14.1 (11.9–16.2)	
Out-of-pocket costs as a percentage of total household expenditure	238		36.5% (27.4–45.5)	
Proportion of households with catastrophic expenditures	142		25.4 (95% CI 18.4–33.3)	

## Discussion

Our findings show that 78% of caretakers paid user fees for inpatient paediatric care for sick children. The mean out-of-pocket costs for inpatient paediatric care were US$ 14.1 (95%CI 11.9–16.2), which is almost three times higher than reported out-of-pocket costs for district hospital paediatric inpatient care in Tanzania (US$ 5.5) (Saksena *et al.* 2010). Given that children under 5 years of age are officially exempted from user fees in public health facilities in Kenya, it is apparent that this policy is not well implemented in practice. The violation of the user fee exemption policy in Kenya has been reported in previous studies (Ayieko *et al.* 2009; Chuma *et al.* 2009), with funding gaps given as a reason for this violation (Chuma *et al.* 2009). The poor implementation of exemption and waiver mechanisms within cost-sharing policies is likely to introduce inequities in access to child health.

The mean percentage of out-of-pocket costs to total household expenditure on essentials was 36.5% (95% CI 27.4–45.5). This level of healthcare expenditure is arguably high and comparable to findings in Tanzania of 35.4% (Saksena *et al.* 2010). A cost burden >40% of household non-subsistence expenditure is likely to be catastrophic to the household (Xu *et al.* 2003). Based on this definition, out-of-pocket expenditures on health were catastrophic for 25.4% (95% CI, 18.4–33.3) of caretakers interviewed. The mean percentage of out-of-pocket costs to non-subsistence household expenditure was 46.9% (95% CI, 28.5–65.2). When households were divided into 2 socio-economic groups, catastrophic costs were incurred by 41.3% (95% CI, 29.0–54.4) in the lower socio-economic group and 11.5% (95% CI, 5.4–20.8) in the higher socio-economic group. As expected, out-of-pocket expenditures in these households appear to be regressive with a greater burden being experienced by households in lower socio-economic groups given that their capacity to pay is diminished compared to households in higher socio-economic groups (Figure 1).

This study has a number of limitations. Data on household monthly expenditures depend on recalled expenditure over a period of a month and are subject to recall biases. Out-of-pocket costs reported are lower than actual costs, given that we did not collect information on other direct costs (e.g. expenses for food and upkeep of any accompanying relatives). The study also only focused on out-of-pocket costs associated with hospital admissions and left out costs associated with outpatient visits and with those who did not seek care in hospitals; these costs would be important in giving a comprehensive picture of out-of-pocket healthcare costs associated with child illnesses. Also, whereas catastrophic health expenditures are conventionally calculated based on annual health expenditure and annual consumption expenditure, the data available allowed for a calculation based on monthly household and health expenditures. These limitations notwithstanding the data presented are potentially useful as inputs in costing and/or cost-effectiveness models that require patient cost and suggest there are significant-out-of pocket costs associated with paediatric admission care in district hospitals in Kenya, which offer a barrier to access to care.

Policy makers need to mitigate the adverse effects of such expenditures, for example, by extending insurance coverage to the uninsured or by increasing efforts to implement exemption mechanisms. Our findings reinforce observations of tension between policy makers and health facility managers in resource-limited settings, with managers trying to provide services with limited resources and hence forced to disregard policies that threaten their revenue streams.
